# Generation of Nucleic Acid Aptamer Candidates against a Novel Calicivirus Protein Target

**DOI:** 10.3390/v13091716

**Published:** 2021-08-29

**Authors:** Jeremy Faircloth, Matthew D. Moore, Sloane Stoufer, Minji Kim, Lee-Ann Jaykus

**Affiliations:** 1 Department of Food, Bioprocessing, and Nutrition Sciences, North Carolina State University, Raleigh, NC 27695, USA; jpfaircl@ncsu.edu (J.F.); lajaykus@ncsu.edu (L.-A.J.); 2 Department of Food Science, University of Massachusetts, Amherst, MA 01003, USA; sstoufer@umass.edu (S.S.); mkim0@umass.edu (M.K.)

**Keywords:** norovirus, aptamer, VPg, detection, Norwalk virus, Tulane virus, infectivity, therapeutics

## Abstract

Human norovirus is the leading cause of foodborne illness globally. One of the challenges in detecting noroviruses is the identification of a completely broadly reactive ligand; however, all detection ligands generated to date target the viral capsid, the outermost of which is the most variable region of the genome. The VPg is a protein covalently linked to the viral genome that is necessary for replication but hitherto remains underexplored as a target for detection or therapeutics. The purpose of this work was to generate nucleic acid aptamers against human norovirus (Norwalk) and cultivable surrogate (Tulane) VPgs for future use in detection and therapeutics. Eight rounds of positive-SELEX and two rounds of counter-SELEX were performed. Five and eight unique aptamer sequences were identified for Norwalk and Tulane VPg, respectively, all of which were predicted to be stable (∆G < −5.0) and one of which occurred in both pools. All candidates displayed binding to both Tulane and Norwalk VPg (positive:negative > 5.0), and all but two of the candidates displayed very strong binding (positive:negative > 10.0), significantly higher than binding to the negative control protein (*p* < 0.05). Overall, this work reports a number of aptamer candidates found to be broadly reactive and specific for in vitro-expressed VPgs across genus that could be used for future application in detection or therapeutics. Future work characterizing binding of the aptamer candidates against native VPgs and in therapeutic applications is needed to further evaluate their application.

## 1. Introduction

Human noroviruses are the leading cause of foodborne illness globally [1]. There are significant challenges related to control of human noroviruses due to a number of viral characteristics. Human noroviruses have a low infectious dose and can persist in the environment for long periods, making sensitive, portable detection in food and environmental samples crucial for control of this highly transmissible virus [2]. Generally, detection of noroviruses involves either (i) genomic amplification or (ii) ligand-based detection techniques. The former has the advantage of being highly sensitive but lacks portability and can take over 1.5 h. Ligand-based detection techniques (like lateral flow assays) can be portable and relatively rapid; however, these techniques have several limitations for norovirus, largely due to a lack of broadly sensitive ligands for the wide range of norovirus genogroups [3].

Human noroviruses are very diverse—especially in the sequence/structure of their proteinaceous capsid, encoded by the second open reading frame of the viral genome. In fact, the outermost region of the capsid protein sequence is the most variable segment of the norovirus genome [2]. This creates a challenge when conventional ligands, such as monoclonal antibodies, are used because the antibodies generally lack cross-reactivity with all viral strains [4,5,6]. Alternatively, carbohydrates known as “histo-blood group antigens” have been used as ligands for the virus, as these carbohydrates are suspected to be co-receptors for a number of human norovirus strains. Histo-blood group antigens are also better able to specifically bind infectious (functional) virus particles compared to antibodies, a number of which can bind structurally compromised viral capsid protein. This is advantageous because in vitro cultivation methods for human noroviruses cannot realistically be utilized for routine food and environmental detection [7,8]. However, a number of norovirus strains have been reported not to bind any histo-blood group antigen, thus limiting their applicability as a truly cross-reactive recognition ligand for noroviruses [9,10,11,12,13,14]. 

Nucleic acid aptamers are a group of alternative ligands composed of single-stranded nucleic acid (ssDNA or RNA) that fold into unique 3D structures determined by their sequence. Because they are single-stranded nucleic acids, aptamers have a number of advantages over the conventional ligands discussed above in that they are: highly stable, easily synthesized, easily purified and modified, relatively inexpensive, do not invoke an adaptive immune response, and require only knowledge of the sequence to produce/utilize [15,16,17]. A number of aptamers have been generated against human and murine noroviruses [5,18,19,20] with promising results for their utilization as detection ligands for noroviruses. Additionally, Moore et al. [21] demonstrated that at least one of the broadly reactive aptamers exhibited binding behavior dependent on capsid stability similar to histo-blood group antigens, meaning that aptamers have the potential to better discriminate infectious particles from non-infectious particles. However, all of these reported norovirus aptamers have been generated against the variable norovirus capsid protein and thus do not display strong reactivity for a number of norovirus genotypes/strains that are structurally more distinct than the strain against which they were selected [18,19,21]. 

In addition to the viral capsid proteins, a protein known as VPg, or viral protein genome-linked, is covalently linked to the 5′ end of the norovirus genome and encapsulated in infectious viral particles. The VPg is involved in recruiting host translation of viral genomic and subgenomic RNA as well as in viral genomic replication [22,23,24]. Further, it is more sequentially conserved than the viral capsid protein and also is necessary for viral replication [25], thus making it an interesting target for broad norovirus detection as well as therapeutics. The purpose of this study was to generate ssDNA aptamers against the VPg of a human norovirus (GI.1 Norwalk, the prototype strain), as well as a cultivable norovirus surrogate (Tulane virus) expressed *in vitro* in *Escherichia coli*. Both a human norovirus and cultivable surrogate were targeted in order to enable subsequent study (beyond the scope of this work) of the generated aptamer candidates for their use in discrimination of infectious viral particles as well as therapeutics. 

## 2. Materials and Methods

### 2.1. Cloning and Expression of VPgs

De-identified Norwalk virus (GI.1; NV) clinical stool specimens were kindly provided by C. L. Moe (Emory University, Atlanta, GA, USA). Tulane virus (TV) and rhesus monkey kidney (LLC-MK2) cells were kindly provided by Xi Jiang (Cincinnati Children’s Hospital Medical Center, Cincinnati, OH, USA). TV was cultured as previously reported [26,27] and a lysate containing viral capsid was obtained via serial freeze-thaw cycles and filtration with a 0.2 µm filter. Both viral stocks were stored in low volume aliquots at −80 °C until use.

Genomic RNA from both viruses was extracted using a viral extraction kit (Qiagen, Germantown, MD, USA) and then reverse transcribed using the Ambion RETROScript kit (Waltham, MA, USA) with 5 µM of primers designed against the VPg region of the cDNA (Table 1) and product cleaned with a Qiagen Qiaquick kit. Primers for NV were taken from previously performed work [28] and are listed in Table 1 below. TV primers were designed using the genome and cleavage sites estimated by Farkas et al. [26] and are also listed in Table 1 below. The VPg sequences were amplified with PCR, product cleaned using a Qiagen Qiaquick kit, and digested with *Eco*RI and *Xho*I restriction enzymes. Sequences were ligated into digested pGEX 4T-1 plasmid vector (GE Healthcare Life Sciences, Chicago, IL, USA) using an Epicentre Fast-Link kit (Lucigen, Middleton, WI, USA) at a 2:1 insert:vector ratio then electroporated into *Escherichia coli* BL21(DE3) cells (Lucigen E. cloni electrocompetent cells). The pGEX-4T-1 vector contains an N-terminal glutathione-S-transferase (GST) protein tag that can be used to anchor the VPg protein to Sepharose beads for aptamer selection. Successful product was confirmed via sequencing (Genewiz Inc., South Plainfield, NJ, USA). Cloned GST-VPg was overexpressed by diluting an incubation of overnight cells 1:10 into fresh 2x YT-ampicillin broth and incubating at 37 °C with shaking until OD_600_ = 0.6–0.9, after which isopropyl β-d-1-thiogalactopyranoside (IPTG) was added to a final concentration of 1 mM. Samples were incubated overnight with shaking at 25 °C. The cells were then pelleted and lysed using a bead beater with acid-washed beads (Sigma, St. Louis, MO, USA) followed by centrifugation and reservation of the supernatant as previously described [5]. Supernatant containing GST-VPg was aliquoted into one-time use aliquots and stored at −80 °C until used in the SELEX process. Confirmation of overexpression of the constructs was confirmed using an anti-GST monoclonal primary antibody (Invitrogen, Waltham, MA, USA, #13-6700) and horseradish peroxidase (HRP)–conjugated anti-mouse IgG secondary antibody (Sigma, St. Louis, MO, USA, #A3562) for Western blotting of the lysate supernatant. 

### 2.2. Aptamer Selection for Human Norovirus and Tulane Virus VPgs

The aptamer selection process for GST-VPg was conducted as previously reported [5] except with GST-VPg protein target as opposed to the P domain of the major capsid protein. 

#### 2.2.1. Preparation of the ssDNA Library

An 81 nucleotides library containing 40 nucleotide variable regions was obtained from IDT Inc. (Coralville, IA, USA). A biotinylated dsDNA library was generated using PCR with aptamer constant region primers (Table 1), with a biotinylated reverse primer. The biotinylated dsDNA library was then bound to streptavidin-coated paramagnetic particles (MagneSphere, Promega, Madison, WI, USA) and captured by magnet. The complementary strand was denatured and washed away using 0.15 M sodium hydroxide and washed thrice with Tris-EDTA (TE). The remaining immobilized, biotinylated strand was then removed from the paramagnetic particles via incubation with 28% ammonium hydroxide at 85 °C for 10 min. Residual ammonium hydroxide was removed using Vivaspin 500 filters with a 10,000 Dalton molecular weight cut-off (Sartorius Stedim Biotech, Cedex, France) and two washes of nuclease-free water. The biotinylated ssDNA library was immediately stored in −80 °C until use [5]. 

#### 2.2.2. Preparation of GST-VPg Protein Target

Positive systematic evolution of ligands by exponential enrichment (SELEX) was performed against GST-VPg (Tulane or Norwalk VPg) by incubating GST-VPg lysate supernatant 1:1 (*v/v*) with 50% glutathione Sepharose 4B bead solution (GE Healthcare, Little Chalfont, UK) at 23 °C for 45 min with flipping followed by washing. For counter-SELEX, a construct containing only the GST tag was overexpressed and GST-tagged beads were prepared as described above. Additionally, human stool confirmed negative for human norovirus was incubated with the beads to remove sequences binding the components of stool. 

#### 2.2.3. SELEX and Counter-SELEX Process

Overall, eight rounds of positive SELEX and two rounds of counter-SELEX were performed as previously described [5,18]. Specifically, approximately 500 pmol of the library prepared above was heated at 90 °C for 10 min followed by 10 min of cooling on ice. For counter-SELEX, the prepared library was incubated with a 125 µL bead bed volume of GST-negative stool beads for 1 h at 23 °C with flipping. The solution was centrifuged at 500× *g* for 5 min and supernatant reserved to capture the non-binding aptamer sequences in the pool. The reserved supernatant was then purified via phenol-chloroform-isoamyl alcohol extraction and ethanol precipitation with coprecipitant (Ambion GlycoBlue, Life Technologies, Grand Island, NY, USA). The purified pellet was reconstituted with 25 µL nuclease-free water. Concentration was reduced to 20–40 ng/mL if needed and 2 µL of the purified DNA was used as template in 50 µL PCR reactions with the aptamer constant region primers in Table 1. The reactions contained 1x GoTaq buffer (Promega, Madison, WI, USA), 500 nM of each primer, 0.2 mM PCR nucleotide mix (Promega, Madison, WI, USA), 0.5 µg single-stranded binding protein (Promega, Madison, WI, USA), and 2 U of GoTaq polymerase (Promega, Madison, WI, USA). Cycling with an initial 95 °C step for 2 min was followed by 30 cycles of 95 °C for 30 s, 50–65 °C for 30 s, and 72 °C for 15 s, followed by a single final step of 72 °C for 5 min. After every SELEX and counter-SELEX round, an initial annealing gradient from 50–65 °C was performed prior to large scale amplification of the library. After large scale amplification of the library using multiple reactions under the optimal annealing temperature, the PCR product was phenol-chloroform extracted with ethanol precipitation to generate concentrated product. The product was then subjected to the same procedure described above to generate a biotinylated ssDNA aptamer pool. 

An initial round of counter-SELEX was performed first to initially clarify any nonspecifically binding sequences, followed by eight rounds of positive SELEX. Positive SELEX was performed using the same procedure as described above, except beads were bound with GST-VPg protein target. After incubation with the pool, the unbound sequences in the supernatant were discarded followed by washing. Then, the bound aptamer-GST-VPg complexes were eluted from the beads via incubation with glutathione elution buffer (50 mM Tris-HCl/10 mM reduced glutathione buffer, pH 8.0) followed by phenol-chloroform extraction and ethanol precipitation. 

After all eight rounds of positive SELEX, one final round of counter-SELEX using GST lysate and norovirus-negative human stool was performed prior to sequencing. For sequencing, the remaining pool was amplified as described above and resolved on 2% agarose gel. The amplification product was purified from the gel using the QIAquick Gel Extraction Kit (Qiagen, Hilden, Germany). The purified pool was then cloned via the TOPO^®^ TA Cloning Kit with electroporation (Invitrogen, Waltham, MA, USA). Colonies containing the cloned aptamer sequences were selected, grown, plasmid extracted, and screened via PCR using the M13 primer set. Colonies that contained plasmid with aptamer insert were sent off for sequencing using the M13 primer set (Genewiz, Inc., Cambridge, MA, USA).

#### 2.2.4. Analysis of Aptamer Sequences, Structural Folding, and Stability

Obtained aptamer sequences (Table 2 and Table 3) were grouped into identical sequences and the proportion of the overall usable aptamer sequence pool was determined. Further, secondary structure and ∆G prediction of each sequenced aptamer were determined using the DNAMfold online server with structures meant to reflect an analytical environment in phosphate-buffered saline (PBS), with 137 mM Na^+^ at 23 °C (http://www.unafold.org/mfold/applications/dna-folding-form.php, accessed on 12 November 2018) [30]. The unique sequence regions of each of the pools were further subjected to motif analysis using the MEME Suite online server (http://meme.nbcr.net/meme/tools/meme, accessed on 14 November 2018) with a minimum motif length of 6 nucleotides and no more than two base mismatches [5,31,32].

#### 2.2.5. Overexpression of VPg Proteins

Separate cultures for GST-VPg (Norwalk virus), GST-VPg (Tulane virus), and GST-only were grown overnight at 37 °C in 2x yeast extract tryptone media with 100 µg/mL ampicillin (2x YTA). Cultures were centrifuged at 5000× *g* for 5 min and cells resuspended in fresh 2x YTA and used to seed 100 mL cultures. The larger cultures were incubated at 37 °C in a shaking incubator until OD_600_ of 0.6–0.7 followed by induction with 1 mM IPTG. After induction, cultures were incubated for ~20 h at room temperature with gentle shaking. Following expression, the cultures were pelleted at 7700× *g* for 10 min at 4 °C and pellets resuspended using 50 µL PBS per mL of culture. Resuspended cells were stored at −80 °C in 2.5 mL aliquots for later sonication.

#### 2.2.6. Preparation of Crude Extracts

Sonication was performed with a Digital Sonifier 450 (Branson Ultrasonics, Danbury, CT, USA) using 60% amplitude setting and cycling 10 s on, 20 s off. Samples were kept on ice during the full sonication procedure, repeating the cycling 4–8 times or until partial clearing of sample. To aid fusion protein solubility, Triton X-100 (Sigma-Aldrich, St. Louis, MO, USA) was added to the sample at a final concentration of 1% and samples were incubated at 4 °C on a rotator for 30 min. Crude extracts were generated by recovering the supernatant following centrifugation at 12,000× *g* for 10 min at 4 °C to remove insoluble material. Crude extracts were made new on the day of any experiment and stored at 4 °C until use. Proper overexpression of all three constructs was confirmed using Coomassie staining and Western blotting as described above with anti-GST monoclonal primary antibody (Invitrogen, Waltham, MA, USA, #13-6700) and horseradish peroxidase (HRP)–conjugated anti-mouse IgG secondary antibody (Sigma, St. Louis, MO, USA, #A3562) for Western blotting of the lysate supernatant. To our knowledge, primary antibodies raised against norovirus VPg proteins were/are not available; however, sequencing and expected protein size via Western blotting suggest proper overexpression of VPg proteins (Figure S1). Future work further characterizing the binding of the generated aptamers against native protein would be a logical next step.

#### 2.2.7. Enzyme-Linked Aptamer Sorbent Assay (ELASA)

An ELASA was used to determine relative binding affinity of each aptamer to both Norwalk and Tulane virus VPg proteins based on previously reported procedures [5,18,33], using glutathione coated 96-well plates pre-blocked with bovine serum albumin (BSA) and SuperBlock buffer (Thermo Scientific, Waltham, MA, USA). Crude protein extracts for GST-VPg (Norwalk virus), GST-VPg (Tulane virus), and GST-only were diluted to 1% solutions in PBS with 0.05% Tween 20 (PBST) prior to use for the binding assay. For each run, aptamers were tested with duplicate wells against all three protein targets and a negative control, and the full experiment was replicated three times.

Glutathione coated plates were rinsed 3 times with 200 µL per well of PBST to remove any residual blocking buffer and incubated with 100 µL per well of diluted crude protein extracts for 1 h at room temperature on an orbital shaker with gentle shaking. Crude protein extracts were removed, and wells were rinsed 3 times with 200 µL PBST to remove any unbound fusion protein and cellular debris. Then, 100 µL of biotinylated aptamer was added to each well at a concentration of 1 mM and plates were incubated on an orbital shaker at room temperature for 1 h. Unbound aptamer was then removed by washing 3 times with 200 µL PBST. To each well, 100 µL of ELISA-grade Streptavidin-HRP (Invitrogen, Carlsbad, CA, USA) solution was added at a final concentration of 1:5000 in PBS, and plates were incubated on an orbital shaker for 15 min. Enzyme conjugate solution was removed and wells washed 4 times with 200 µL PBST. TMB microwell peroxidase substrate (KPL, Gaithersburg, MD, USA) was used for detection, with 100 µL of substrate solution added to each well followed by incubation on an orbital shaker. The reaction was stopped after 3 min by addition of 100 µL of TMB stop solution (KPL). Plates were immediately read on a Tecan Infinite M200pro microplate reader at 450 nm (Tecan Group Ltd., Männedorf, Switzerland).

#### 2.2.8. ELASA Optimization and Validation

Concentrations of crude protein extract and aptamer were optimized prior to testing against the panel of aptamers. GST-VPg (Norwalk virus) and GST-VPg (Tulane virus) extracts were both tested at concentrations of 100%, 10%, and 1% diluted in PBST, using aptamer concentrations of 1 mM. Due to its presence in both Norwalk and Tulane aptamer pools, aptamer T5 (Table 2) was used for optimization of specific binding. Binding affinity was similar across all concentrations for GST-VPg (Tulane virus), but higher at 1% for GST-VPg (Norwalk virus) (Figure S4). Non-specific binding of aptamers was tested using GST-only cultures at 100%, 10%, and 1% with 1 mM aptamer T5. Two additional aptamer sequences of aptamers targeting an unrelated protein that is structurally not similar to VPg (norovirus capsid protein), M6-2 and M6-2 scrambled [5,21], were included to get a more complete understanding of the non-specific binding behavior of the assay (Figure S3). 

Non-specific binding of all three aptamers was similar across all protein extract concentrations tested, with a trend of decreasing absorbance ratio with higher dilution of the protein extract. Based on the results from specific and non-specific binding, a dilution of 1% was used for the crude protein extracts in the final binding affinity assay. Aptamer concentration was optimized using aptamer T5 at concentrations of 0.01, 0.05, 0.2, 0.5, and 1 mM, against a 1% dilution of GST-VPg (Tulane virus). The highest positive:negative ratio was provided by 1 mM, and this was chosen for subsequent affinity assays (Figure S2).

#### 2.2.9. Binding Affinity Analysis

A total of 12 aptamer sequences were examined, 7 developed against Norwalk virus, 4 against Tulane virus, and 1 common to both. GST-only controls were added for each aptamer to determine degree of non-specific binding to either the GST tag or other objects present in the crude protein extract as described above. Absorbance ratios of the overall positive absorbance divided by PBS-only (no lysate/protein) wells for Norwalk VPg, Tulane VPg, and GST lysate were determined, with a positive:negative absorbance ratio >2 indicating positive signal, per convention [18,34,35]. Negative controls containing PBS-only (no protein/lysate) wells as well as GST-lysate wells were used as above. At least two wells per sample were used for each plate, with at least three plates replicated on separate days. Statistical analysis was performed via ANOVA using R. Post-hoc comparison of means was performed using Tukey’s HSD, with a *p* value of <0.05 considered statistically significant.

## 3. Results

### 3.1. Aptamer Candidates

After two rounds of counter SELEX and eight rounds of positive SELEX against the Norwalk virus (GI.1) and Tulane virus VPg proteins, a number of different pools of aptamer sequence for each target were obtained and sequenced (Table 2 and Table 3). Five and eight unique sequences were obtained in pools of 17 and 19 total obtained sequences for Tulane and Norwalk VPgs, respectively. The Tulane VPg pool was less diverse than the Norwalk and consisted mostly of one sequence, T5, which comprised about 76% of the sequence pool. The Norwalk VPg pool had one sequence that comprised about 47% of the pool, but a number of additional unique sequences were also identified. Interestingly, the one sequence that made up the majority of the Tulane pool (T5; Table 2) also was identified once in the Norwalk pool (N12-2; Table 3), suggesting the potential for some cross-reactivity.

Aptamer pools all would be expected to display relative stability based on predicted ∆G values, which ranged from −5.95 to −13.46 at PBS sodium levels in room temperature—conditions likely to be used in an analytical/sensing setting. Aptamer pool candidates and secondary structures displayed a diversity of structures. MEME analysis for the Norwalk pool found no common significant (defined as a calculated “E-value” <0.05) motifs; however, a significant motif was identified for four of the five unique sequences of the Tulane pool. Specifically, a motif of “CTGACAACCCG” was present starting at around the 21–30th base of T1-2, T9, T9-2, and T10-2. This motif was also largely present on protruding loop regions of all of the predicted secondary structures for the aptamers, suggesting it may be a conserved sequence involved in aptamer binding.

### 3.2. Aptamer Reactivty

**Figure 1:**
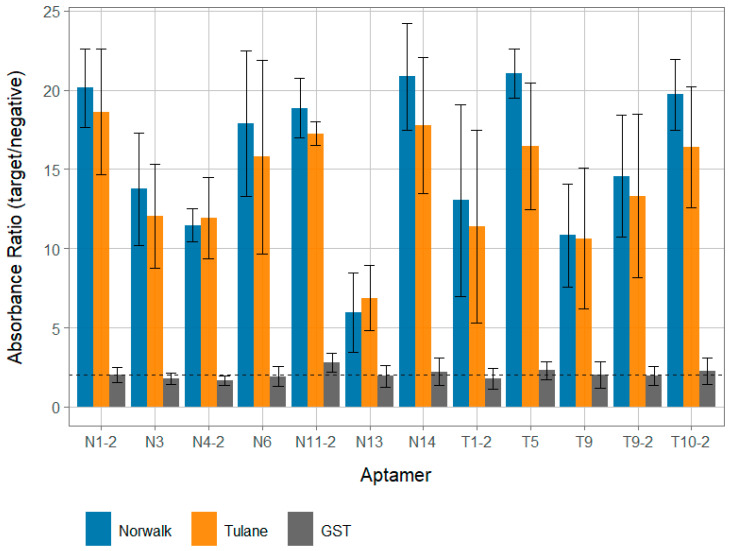
Affinity of aptamer VPg candidates. The relative binding ratios of each of the developed aptamers against Norwalk (blue) and Tulane (orange) VPg proteins were analyzed for each aptamer candidate. Further, binding to GST protein for each is visualized with grey bars. All ratios represent the ratio of positive absolute absorbance to absorbance for PBS only (no protein) wells. Aptamers that begin with “N” were generated against the Norwalk VPg and aptamers with a “T” in their name were generated against the Tulane VPg. Per convention, a ratio <2.0 is little to no binding, 2.0–5.0 low binding, 5.0–10.0 moderate to strong binding, and >10.0 very strong binding [5,18,34,35].

All 12 of the identified aptamer candidates demonstrated reactivity with both Tulane and Norwalk VPg targets, with postive:negative absorbance ratios above 5.0 (Figure 1). All aptamer candidates except N13 displayed very strong binding that was significantly (*p* < 0.05) higher than binding to GST, with all average positive:negative ratios greater than 10.0 for both VPg targets. Interestingly, aptamers displayed strong binding to both the VPg they were generated against and the other VPg (i.e., an aptamer generated against Tulane VPg strongly reacted with Norwalk VPg). Although not statistically significant, aptamers tended to more strongly react against the Norwalk VPg than Tulane VPg, regardless of which target they were raised against.

### 3.3. Aptamer Specificity

While aptamer candidates displayed strong reactivity against the Tulane and VPg clarified protein lysates, no candidate displayed more than a low degree of binding to a clarified GST lysate (Figure 1). Further, all 12 aptamer candidate binding ratios for both VPg targets were statistically significantly (*p* < 0.05), higher for VPg targets compared to GST. This suggests that the observed binding signal is due to the VPg protein for each and not the GST protein or any other component of the clarified lysate. 

## 4. Discussion

The purpose of this study was to generate ssDNA aptamers against a novel calicivirus target protein cloned in *E. coli* and expressed in vitro, the VPg, for others to further utilize and investigate in detection and therapeutic applications. After selection of aptamers against a human norovirus lab type strain (Norwalk, GI.1) and Tulane virus, a cultivable related surrogate virus that replicates in the enteric tract of rhesus macaques [26], 12 unique candidate VPg aptamer sequences were obtained out of a collective pool of 36 usable sequences. All sequences displayed reasonable predicted stability (∆G) in PBS and displayed a number of overlapping motifs. Other previously reported norovirus aptamers generated against the capsid protein all displayed free energies within the range observed here [5,18,19]. Interestingly, a strongly significant sequence motif implicated in binding was identified in the Tulane aptamer pool. Further, it was present on 4/5 of the Tulane sequences, but not the one with the disproportionately high proportion of the pool that was also present in the Norwalk pool (T5, Table 2). Given the fact that all four of the sequences displayed reactivity with Norwalk VPg expressed in vitro, this motif may be implicated in binding for both VPgs.

Nearly all aptamer candidates displayed very strong binding to both Tulane and Norwalk VPg proteins cloned into *E. coli* and expressed in vitro in clarified protein lysates (positive:negative ratios > 10.0), regardless of which VPg the candidate was selected against (Figure 1). It should be noted that all binding data from this work was performed against the in vitro VPg against which the aptamers were generated; further confirmation of aptamer candidate binding against native VPg isolated from infectious viruses (Tulane and Norwalk) was not able to be conducted due to limitations in funding, personnel, and availability of reagents. However, GST-VPg cloned and expressed in *E. coli* does seem to display a similar functionality as native VPg in its ability to bind mammalian host translation factors for both Norwalk virus and murine norovirus [28,36], though the potential for different post-translational modifications of native VPg that would alter aptamer candidate binding behavior still exists. These positive:negative values are largely at the higher end of previously reported values observed for aptamers targeting the norovirus capsid that were also evaluated using a similar assay with approximately the same concentration of target [5,18], though exact concentration was not measured for the purposes of demonstrating binding. Conversely, all aptamer candidates displayed very minimal reactivity to GST clarified protein lysate; suggesting that aptamer candidates were specific for the VPg protein and not GST or other associated proteins that may be present in clarified lysate. This is further supported by the fact that little binding signal was observed when even higher concentrations (as much as 2 log higher levels; Figure S4) of GST clarified lysate were used against the aptamer that displayed one of the highest levels of binding for the GST lysate (T5; Figure 1). Further, similarly low levels of binding to GST were observed with both a scrambled aptamer and aptamer specific for an unrelated protein target (Figure S3), suggesting that the ELASA assay conditions were stringent enough to remove the influence of most nonspecific binding. Additionally, the predicted free energies for these aptamers were within the range of all other reported human norovirus aptamers, suggesting a reasonable degree of stability [5,18,19,20]. One notable exception of the candidates was aptamer N13, which still displayed reasonable binding, but not as strong as the other candidates (Figure 1). Further, this sequence actually occurred more frequently in the aptamer pool than some of the other candidates (Table 3). The occurrence of sequences in aptamer pools that can still be enriched in SELEX pools is not uncommon, and it is possible that the discrepancy between the proportion of N13 in the pool and its apparent binding affinity could be due to artifacts in the selection process. For example, the anchoring of VPg to GST associated with the bead selection matrix could have altered its structure compared to that of the structure displayed in an ELASA format, especially as both the terminal ends of the VPg are known to be relatively disordered [37]. However, future confirmation of the binding behavior of N13, as well as all the candidates reported here, both from infectious virus and in cellular conditions, should be conducted in the future. In sum, this evidence suggests that many of the aptamer candidates are both specific and have a high level of affinity for VPgs. Although not significantly different (*p* > 0.05), a general trend of higher binding signal with Norwalk VPg compared to Tulane was observed for most aptamers. This could be due to a number of factors, but the most likely is that there was a slightly higher concentration of VPg in the Norwalk preparation compared to the Tulane VPg, which could have been due to more efficient overexpression or effective concentration. The purpose of the binding assays was to demonstrate that specific binding was occurring for the aptamer sequences for others to utilize, not to determine the exact binding affinity or compare binding between VPg proteins. Future work should focus on further characterizing the specific affinity and regions of aptamer responsible for binding. 

All generated aptamer candidates displayed similarly strong degrees of signal for both Tulane and Norwalk VPgs that were expressed in vitro, regardless of which protein they were generated against. This suggests that these aptamers are likely broadly reactive against a wide array of strains in the *Caliciviridae* family, which also contains other human pathogens of concern. This may be expected, as the VPg protein present in infectious capsids is needed for replication and typically more conserved than the variable major capsid protein targeted by many traditional detection ligands (Figure S5) [22,25,37]. This is promising news for both detection and therapeutic applications, as one of the major obstacles to detection for these viruses has been the ability to generate a ligand with broad specificity for the entire diverse group of noroviruses [4,5,6]. Strong binding to both a VPg from the genus *Norovirus* as well as the proposed genus *Recovirus* further suggests the promise of the VPg protein as both a detection target and a therapeutic target for public health. Further work should be conducted to better elucidate the degree to which these aptamers bind the VPgs of other caliciviruses, including murine norovirus (another cultivable surrogate in genogroup V) and other genotypes of human noroviruses. Further, the scope of this research note is narrowed just to confirm specificity of aptamer binding to crudely purified VPg targets overexpressed in *E. coli*; future work to confirm binding with native, virus-associated VPg should also be investigated in the future—both VPg associated with viral RNA and without (RNase-digested) RNA. 

This work also has implications for detection applications, as VPg in virus particles is attached to subgenomic RNA, which may be present in clinical samples. One could potentially increase the analytical sensitivity by coupling VPg capture with RT-qPCR targeting ORF2 or ORF3. Alternatively, one of the major challenges in virus detection is being able to discern whether or not a detected viral genetic sequence corresponds to an infectious virus [7,38]. In order to remove some of the noninfectious RNA, a capture assay utilizing a suspected viral co-receptor is routinely performed, but this still does not completely compensate for amplification of noninfectious particles [7,38]. This creates many challenges in virus detection and study of viral inactivation, as multiple subtypes of caliciviruses—including human noroviruses—do not have an easily adoptable tissue culture assay to determine infectivity, although two breakthrough systems valuable for studying pathogenesis have been reported [39,40]. Previous work by Moore et al. [21] has demonstrated that aptamers targeting the norovirus capsid behave similarly in binding preference to receptors for determination of viral capsid integrity. Future work combining a capsid integrity binding step followed by use of one of the aptamers reported here to account for VPg integrity may remove additional noninfectious particles, as the possibility exists for capsid integrity/functionality to still be maintained while VPg is inactivated/nonfunctional. Previous work has demonstrated that the VPg is required for successful replication in cells, so VPg functionality is likely a better indicator of viral infectivity [25].

Although beyond the scope of this work, the developed aptamer sequences also have the potential to be investigated as therapeutics. This is because the VPg protein is involved in a number of crucial functions for viral replication [22,24,41] and has been demonstrated to be required for viral replication in cells [25]. Specifically, the VPg has been reported to act as a recruitment factor for host translation machinery for genomic and subgenomic RNA [28,36], serve as a template for primer-free replication [42], and disrupt the host cell life cycle [43]. Inhibition of these functions would likely reduce viral replication. Aptamers are also more likely to disrupt protein function than small molecules due to their size, which makes them advantageous for use as therapeutics [44]. The fact that the sequences reported here seem to be broadly reactive beyond their respective genera indicates that they may be promising candidates as therapeutics for infections caused by norovirus as well as other members of the *Caliciviridae* family that infect humans, like sapoviruses. However, a number of considerations must be made regarding the selection conditions used in this study. Specifically, the buffer and selection conditions were more representative of conditions found in detection, so it is possible that aptamers may not bind as strongly at higher temperature and in cellular buffer conditions. Further, the VPgs in a cell may already be associated with the aforementioned host cell machinery and/or viral RNA, which could sterically disrupt the aptamer-VPg interaction. Presumably, any therapeutic benefit would require aptamers to be delivered/present in neighboring cells that have yet to be infected by virus. Future work further elucidating the potential of these aptamers to both bind in a cellular environment and in the presence of virus-infected cells should be conducted. 

## 5. Conclusions

In sum, this work reports a number of ssDNA aptamer sequences that appear to specifically bind a hitherto unexplored calicivirus target protein with potential detection or therapeutic applications. The aptamer sequences reported here displayed strong apparent binding and did not react with the unrelated GST protein or lysed bacterial proteins from *E. coli*. The aptamers appeared to broadly react with VPgs from two different genera of the *Caliciviridae* family, suggesting the VPg is a promising target for generation of broadly reactive ligands for both detection and therapeutic applications.

## Figures and Tables

**Table 1:** Primers used in this study.

Primer Name	Sequence (5′ to 3′) ^a^	Reference
Tulane VPg Forward	CCGGAATTCGCCAAGGGCAAGACAAAAAGG	This work
Tulane VPg Reverse	CCGCTCGAGCTACTCGTCGTAATAATCATCACTGGG	This work
Norwalk VPg Forward	CCGGAATTCGGAAAGAACAAAGGCAAGACC	[28]
Norwalk VPg Reverse	CCGCTCGAGTTCAAAATTGATCTTTTCATTATAAT	[28]
DNA Aptamer Library	AGTATACGTATTACCTGCAGC-N40-CGATATCTCGGAGATCTTGC	[18,29]
Aptamer Forward Constant	AGTATACGTATTACCTGCAGC	[18,29]
Aptamer Reverse Constant	/Biotin/GCAAGATCTCCGAGATATCG	[18,29]

^a^ Restriction enzyme target sequences are underlined.

**Table 2:** Unique Tulane virus VPg aptamer sequences.

Name	Conserved Sequence ^a^	G (kcal/mol)	Frequency
T5	TCACACTCGTTTCTATTACTAAAACATCGTTCCTTTCAGC	−5.95	13/17
T9	TGGAAGGCGGGAAGATTTTTGGTCGACCTGACAACCCGGT	−10.19	1/17
T1-2	TAGTAACGATTACCAAAATTCTCCCGAGGCTGACAACCCG	−6.47	1/17
T9-2	TCGAGGTATGGCCTTGTCTAGGCGCACCTGACAACCCGGTG	−11.89	1/17
T10-2	TGTCGTTAATTATTCGTGATCTGACAACCCGATCACTCTC	−12.01	1/17

^a^ All sequences are flanked by AGTATACGTATTACCTGCAGC at 5′ end and CGATATCTCGGAGATCTTGC at 3′ end.

**Table 3:** Unique Norwalk virus VPg aptamer sequences.

Name	Conserved Sequence ^a^	G (kcal/mol)	Frequency
N3	AGGGATGTGTTGGATGCATGCCAGGCTTGGTAACATTGTA	−9.90	1/19
N6	CAGAGTTGATGTAAGCTTCGTGTTAGCTCAACTCTTATCG	−8.36	9/19
N13	TCTTCGGTTTAATAAAGTTGGCTAGGAAAGTTTAAAACCG	−7.04	3/19
N14	AGTGGGTGGTGATGAATTCTGGTCGCGCTGACAACCCGCG	−11.90	1/19
N1-2	CGGGTCTCGTCTATGCAGTACTCAAAACGCTTGAGGTACCGA	−11.92	1/19
N3-2 ^b^	CAGAGTTGATGTAAGCTTCGTGTTAGCTTAACTCTTATCG	−7.87	1/19
N4-2	AAGGCTTTTTTAAAGGCTAGGCTTGATAATCGGTTAACTC	−13.46	1/19
N11-2	TGTCGATAAAGTGAGTTAAGTCACCGGCCCGGCCTATTCG	−6.63	1/19
N12-2 ^c^	TCACACTCGTTTCTATTACTAAAACATCGTTCCTTTCAGC	−5.95	1/19

^a^ All sequences are flanked by AGTATACGTATTACCTGCAGC at 5′ end and CGATATCTCGGAGATCTTGC at 3′ end. ^b^ Only one base substitution different than N6. ^c^ Same sequence as T5 in Tulane VPg aptamer pool.

## Supplementary Materials

The following are available online at https://www.mdpi.com/article/10.3390/v13091716/s1, Figure S1: Confirmation of proper protein overexpression, Figure S2: Optimization of aptamer concentration for relative ELASA assay, Figure S3: Determination of the degree of nonspecific binding that occurs with different protein lysate concentrations, Figure S4: Determination of the effect of protein lysate concentration on aptamer binding, Figure S5: Amino acid sequence alignment of Tulane and Norwalk VPg proteins. An alignment of the amino acid sequences of the Tulane and Norwalk VPgs targeted in this work.
