# Supplementary material for: Generation of Nucleic Acid Aptamer Candidates against a Novel Calicivirus Protein Target
Source: Viruses. 2021 Aug 29;13(9):1716. doi: 10.3390/v13091716 (PMC8473235; doi:10.3390/v13091716)
Supplement: Supplementary file 1 [file viruses-13-01716-s001.zip › viruses-1332662-supplementary.pdf]

Supplementary Materials

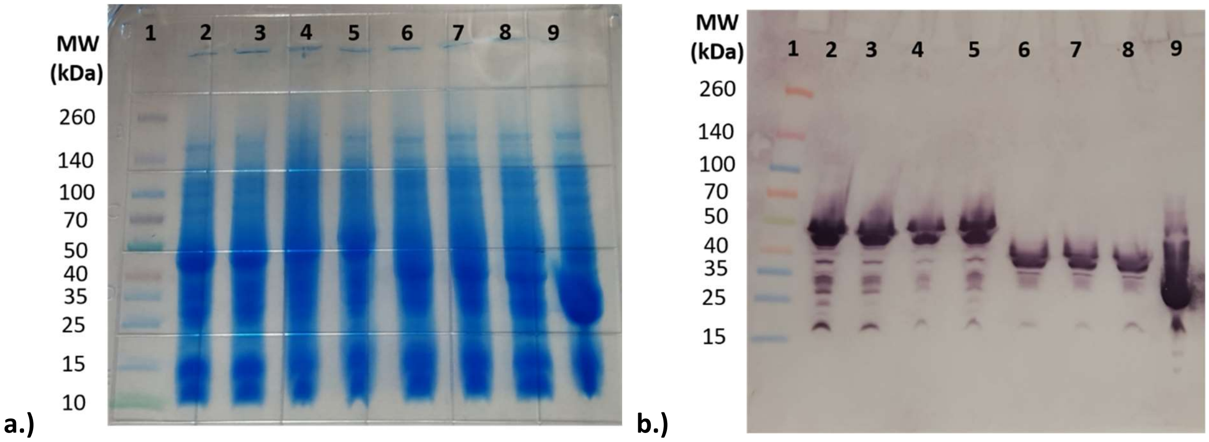

Figure S1. Confirmation of proper protein overexpression. In addition to confirmation of constructs by sequencing, protein overexpression and purification was confirmed using a.) SDS-PAGE protein staining using AcquaGel and b.) Western blotting with anti-GST monoclonal antibody. Lanes for both a.) and b.) reflect the same protein lysate. Lane 1: Ladder; Lanes 2-5: Different colonies of the same GST-Norwalk Virus VPg stock; Lanes 6-8: Different colonies of the same GST-Tulane Virus VPg stock; Lane 9: Colony of a GST-Only stock. Anticipated sizes of the constructs are as follows: GST-Norwalk VPg = 42.5 kDa; GST-Tulane VPg = 37.4 kDa; GST-only = 26 kDa.

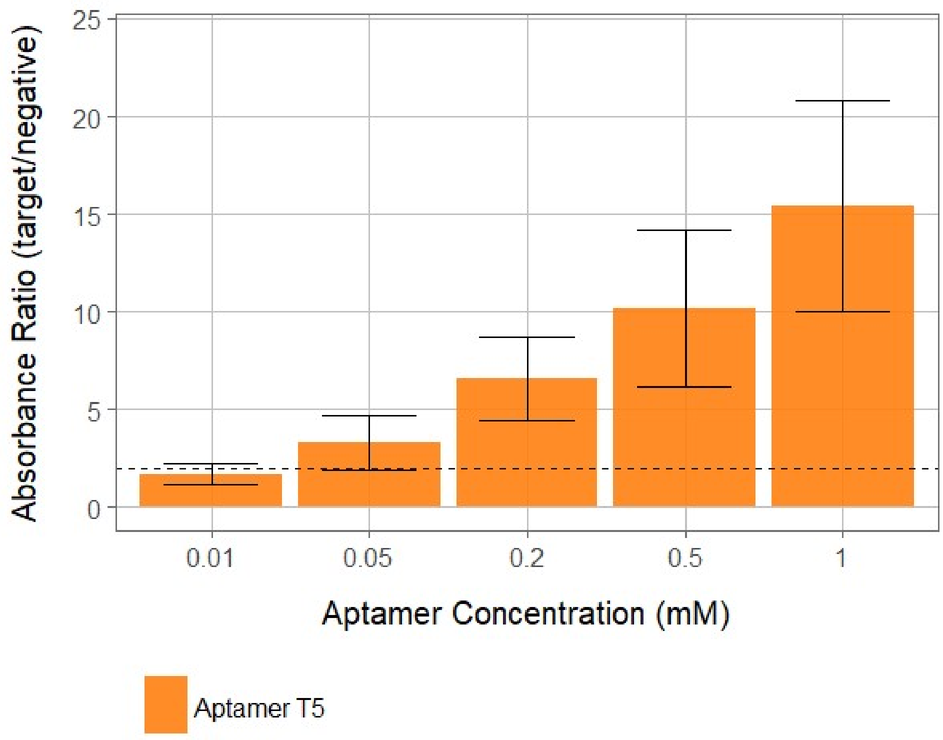

Figure S2. Optimization of aptamer concentration for relative ELASA assay. Different concentrations of a representative aptamer candidate that occurred in both pools, T5, were analyzed against the Tulane VPg protein using an ELASA assay to determine the optimal concentration for use in determining the relative affinity of each of the VPg aptamer candidates.

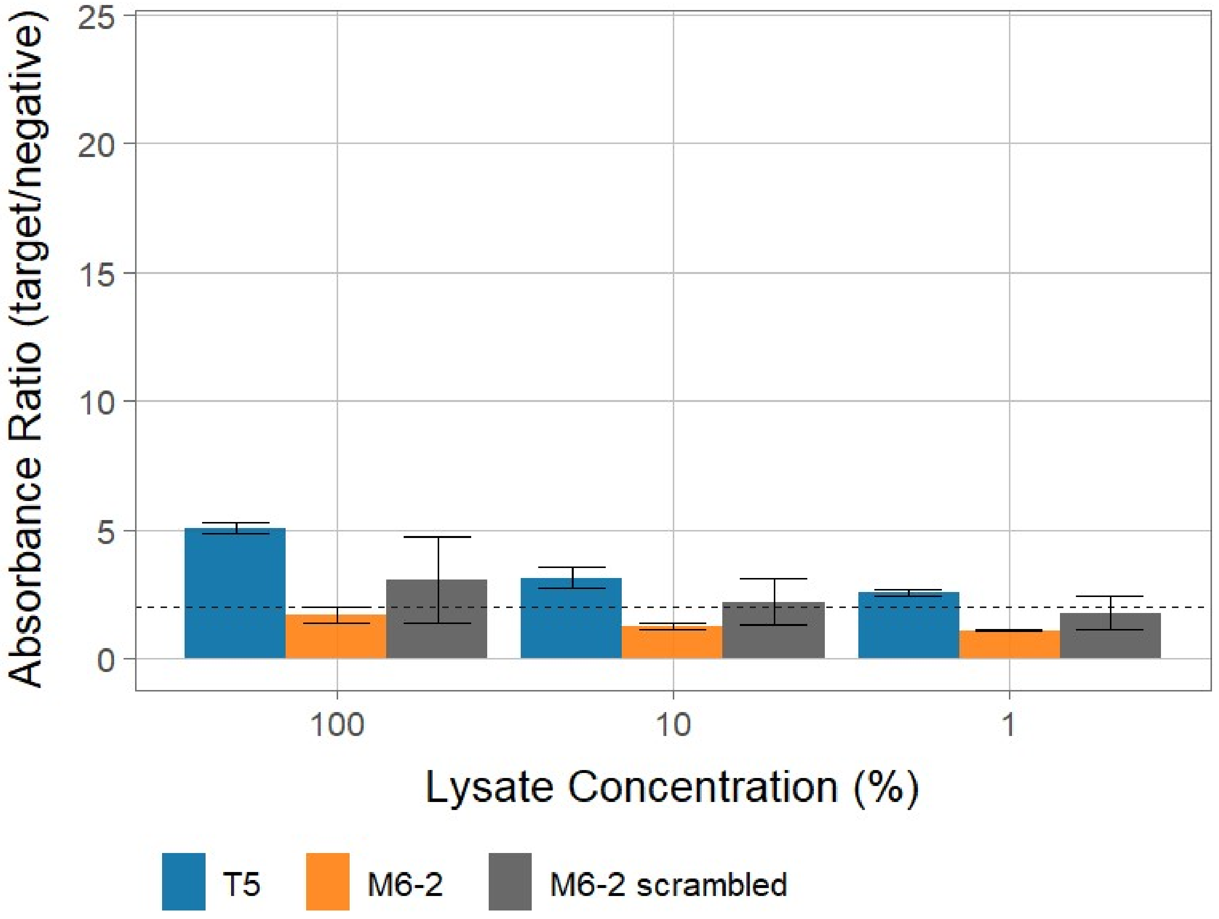

Figure S3. Determination of the degree of nonspecific binding that occurs with different protein lysate concentrations. To assess the relative degree of nonspecific binding that occurs as a function of the protein lysate concentration used in the ELASA assay, three different aptamer sequences were used. One was representative of the current pool (T5), one is an aptamer generated against unrelated protein target that has been demonstrated to be specific [1], and one is a scrambled aptamer sequence. Absorbance ratios are depicted as the ratio of absolute absorbance of protein signal to wells containing PBS only (no protein).

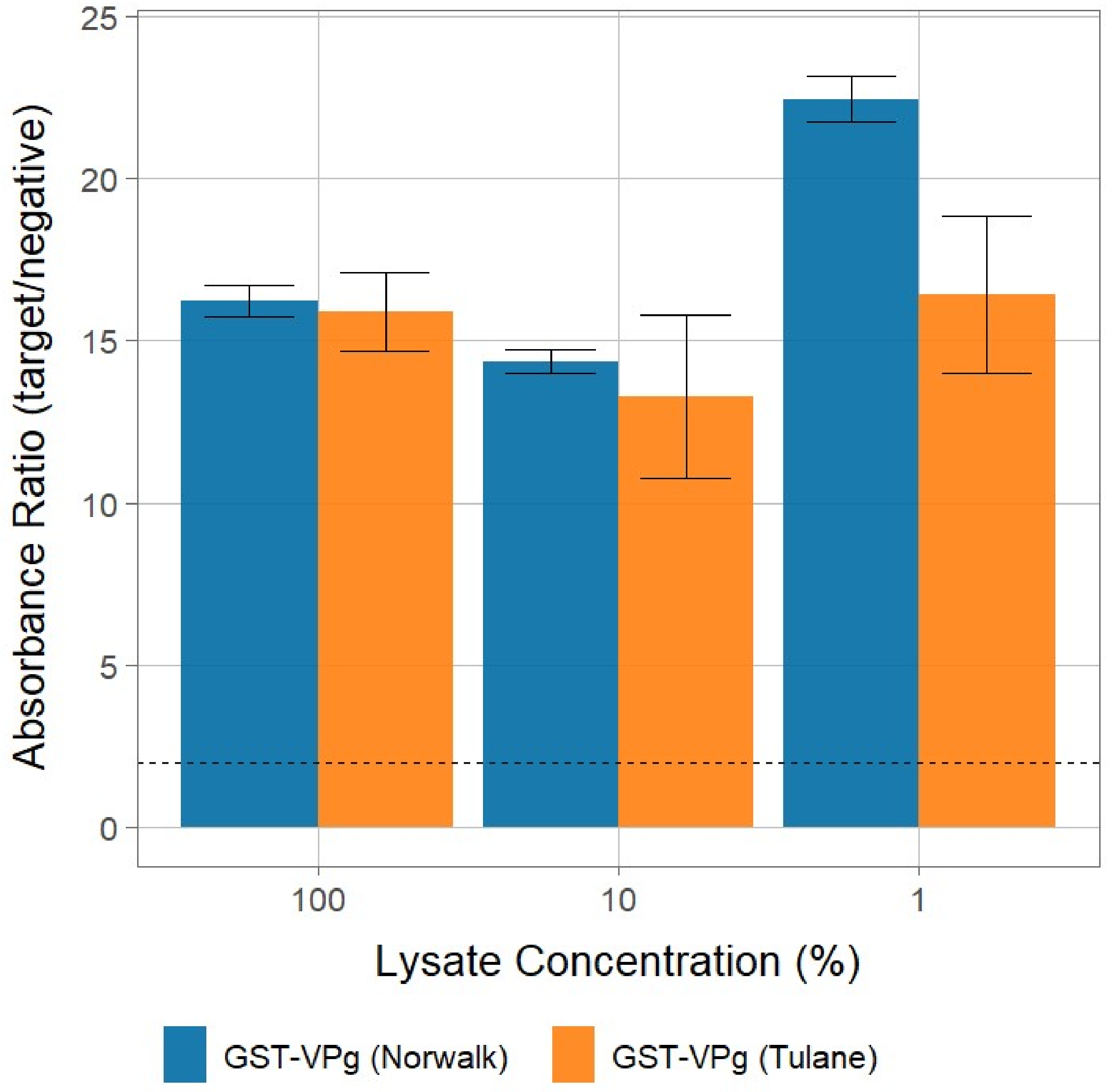

Figure S4. Determination of the effect of protein lysate concentration on aptamer binding. To determine if the protein lysate concentration used at the levels tested caused notable differences in signal, different concentrations of a representative aptamer T5, was used against varying concentrations of Norwalk and Tulane VPg protein lysates. Absorbance ratios are depicted based on the ratio of absorbance for lysate to PBS only (no protein) wells.

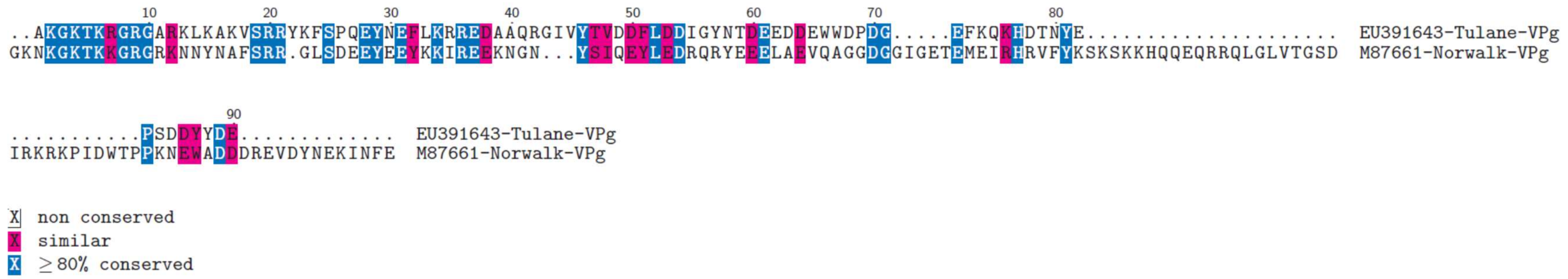

Figure S5. Amino acid sequence alignment of Tulane and Norwalk VPg proteins. An alignment of the amino acid sequences of the Tulane and Norwalk VPgs targeted in this work.

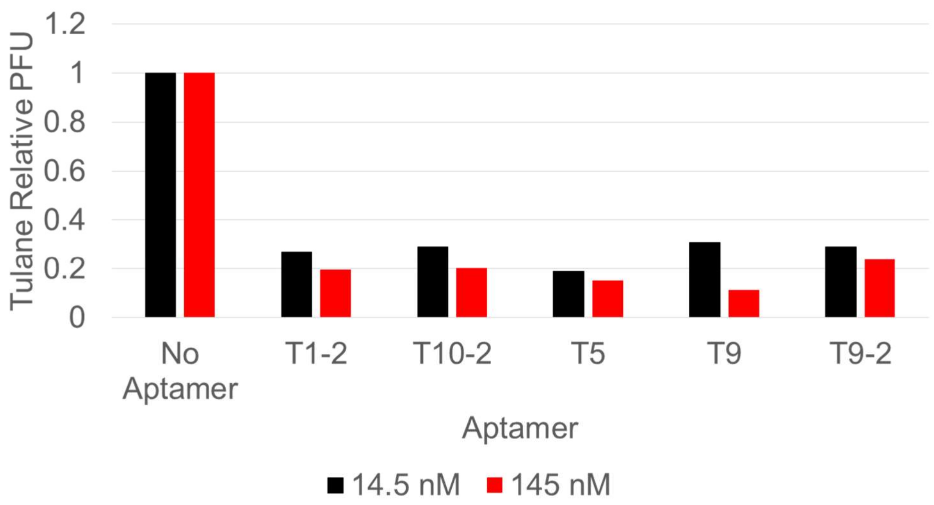

Figure S6. Relative PFU of Tulane Virus after treatment with VPg aptamer candidates. Cells were pretreated with different TV VPg aptamers at 0.5 and 5 nM prior to introduction of TV. The relative PFU (PFU of Treated Samples/PFU of No Aptamer Control) is displayed. Note: controls for viral reduction due to stimulation of innate immunity and/or cytotoxicity were not able to be conducted. Future work should further elucidate any potential influence of these factors on apparent reductions observed, which are comparable to other antiviral aptamer therapeutics [2–4].

Supplemental References

1. Moore, M.D.; Escudero-Abarca, B.I.; Suh, S.H.; Jaykus, L.-A. Generation and Characterization of Nucleic Acid Aptamers Targeting the Capsid P Domain of a Human Norovirus GII.4 Strain. *J. Biotechnol.* **2015**, *209*, 41–49.
2. Afrasiabi, S.; Pourhajibagher, M.; Raoofian, R.; Tabarzad, M.; Bahador, A. Therapeutic applications of nucleic acid aptamers in microbial infections. *J. Biomed. Sci. 2020 271* **2020**, *27*, 1–13.
3. Kim, T.-H.; Lee, S.-W. Aptamers for Anti-Viral Therapeutics and Diagnostics. *Int. J. Mol. Sci. 2021, Vol. 22, Page 4168* **2021**, *22*, 4168.
4. Shi, S.; Yu, X.; Gao, Y.; Xue, B.; Wu, X.; Wang, X.; Yang, D.; Zhu, H. Inhibition of Hepatitis C Virus Production by Aptamers against the. *J. Virol.* **2014**, *88*, 1990–1999.
